# An Exhaustion-Like Phenotype Constrains the Activity of CD4+ T Cells Specific for a Self and Melanoma Antigen

**DOI:** 10.1371/journal.pone.0123332

**Published:** 2015-04-15

**Authors:** Matthew P. Rausch, Karen Taraszka Hastings

**Affiliations:** 1 Department of Basic Medical Sciences, College of Medicine Phoenix, University of Arizona, Phoenix, Arizona, United States of America; 2 Arizona Cancer Center, University of Arizona, Tucson, Arizona, United States of America; 3 Department of Immunobiology, College of Medicine, University of Arizona, Tucson, Arizona, United States of America; MRC National Institute for Medical Research, UNITED KINGDOM

## Abstract

While the immune system has the capacity to recognize and destroy melanoma, tolerance mechanisms often hinder the development of effective anti-tumor immune responses. Since many melanoma antigens are self proteins expressed in normal melanocytes, self antigen exposure before tumor development can negatively impact the function of T cells specific for these self/tumor antigens. However, the contribution of self tolerance to anti-melanoma T cell dysfunction remains largely unexplored. We have previously described a TCR transgenic (Tg) mouse model in which T cells specific for the self/melanoma antigen, tyrosinase-related protein 1 (TRP1), develop in the presence of endogenous TRP1 expression (Ag+) and diminished antigen presentation due to the absence of gamma-interferon-inducible lysosomal thiol reductase (GILT-/-). We show that TRP1-specific T cells from these Ag+GILT-/-Tg mice do not protect from melanoma tumor growth, fail to induce autoimmune vitiligo, and undergo diminished proliferation compared to T cells from Ag-GILT+/+Tg mice. Despite an increased frequency of TRP1-specific Treg cells in Ag+GILT-/-Tg mice compared to Ag-GILT+/+Tg animals, Treg cell depletion only partially rescues the proliferative capacity of T cells from TRP1-expressing mice, suggesting the involvement of additional suppressive mechanisms. An increased percentage of melanoma-specific T cells from Ag+GILT-/-Tg animals express PD-1, an inhibitory receptor associated with the maintenance of T cell exhaustion. Antibody blockade of PD-1 partially improves the ability of TRP1-specific T cells from Ag+GILT-/-Tg mice to produce IL-2. These findings demonstrate that melanoma-specific T cells exposed to a self/melanoma antigen in healthy tissue develop an exhaustion-like phenotype characterized by PD-1-mediated immunosuppression prior to encounter with tumor.

## Introduction

The immune system is capable of recognizing melanoma tumors, and patients readily develop melanoma-specific T cell responses [1, 2, 3, 4, 5, 6]. However, in most cases, these immune responses ultimately fail to eradicate established melanoma tumors. T cells isolated from melanoma-bearing hosts are often characterized by functional impairment [7]. Several mechanisms may contribute to the dysfunction of tumor-specific T cells including 1) tumor antigen encounter during the early premalignant, non-inflammatory phase of tumor development, 2) immunosuppressive factors of the tumor microenvironment, and 3) peripheral T cell tolerance to self antigens [8, 9, 10, 11, 12, 13]. However, the contribution of each mechanism to T cell dysfunction observed in melanoma patients has been difficult to dissect.

Since many of the known melanoma antigens are self proteins expressed in normal melanocytes, it is important to determine the role of self antigen exposure in melanoma-specific T cell dysfunction. Human studies of tumor-infiltrating lymphocytes specific for self/melanoma antigens are unable to assess the impact of self antigen exposure prior to tumor development on T cell tolerance [14, 15, 16, 17, 18]. Animal models of T cells specific for self and melanoma antigens often utilize naïve T cells isolated from self antigen-deficient T cell receptor (TCR) transgenic mice, downplaying the importance of self antigen exposure on T cell dysfunction [19, 20, 21]. Therefore, it is unclear to what extent self antigen exposure prior to tumor development contributes to the functional impairment of T cells specific for self and melanoma antigens.

Our laboratory has developed a mouse model to study mechanisms that constrain CD4+ T cell-mediated immunity to melanoma antigens that are also self antigens [22], using the tyrosinase-related protein (TRP) 1-specific TCR transgenic mouse model generated previously [19]. TRP1-specific T cells are deleted in the thymus of TRP1-expressing RAG1-/- TRP1-specific TCR transgenic mice [19, 22]. However, TRP1-specific T cells escape thymic deletion in TCR transgenic mice that lack expression of either TRP1 or gamma-interferon (IFN)-inducible lysosomal thiol reductase (GILT), an enzyme required for efficient MHC class II-restricted processing of TRP1 [22]. TRP1-specific T cells that develop in TCR transgenic mice lacking TRP1 (Ag-GILT+/+Tg) are naïve, induce autoimmune vitiligo, and have anti-melanoma activity [19, 20, 21, 22]. In contrast, TRP1-specific T cells from TCR transgenic mice expressing TRP1, but lacking GILT expression (Ag+GILT-/-Tg) contain a population of antigen-experienced T cells, have diminished cytokine production, and do not induce autoimmunity [22]. The Ag+GILT-/-Tg mouse model is ideally suited to evaluate the mechanisms that limit melanoma-specific T cell responses in the context of cognate self antigen expression prior to tumor development.

Our laboratory has previously shown that TRP1-specific T cells from Ag+GILT-/-Tg mice fail to induce vitiligo after adoptive transfer in part due to a four-fold increase in the percentage of TRP1-specific Foxp3+ Treg cells in comparison to Ag-GILT+/+Tg mice [22]. While Treg cell depletion partially restores the ability of T cells from Ag+GILT-/-Tg mice to induce vitiligo, Treg cell-depleted melanoma-specific T cells from these animals induce disease with diminished severity and delayed onset in comparison to vitiligo caused by T cells from Ag-GILT+/+Tg mice [22]. Here, we show that Ag+GILT-/-Tg mice are not protected from melanoma tumor growth. In addition, TRP1-specific T cells from Ag+GILT-/-Tg mice underwent diminished antigen-specific proliferation compared to T cells from Ag-GILT+/+Tg mice. The defective proliferative capacity of T cells from Ag+GILT-/-Tg mice persists after Treg cell depletion suggesting that additional mechanisms contribute to the T cell dysfunction in these mice. Since T cells from Ag+GILT-/-Tg mice exhibit many characteristics associated with T cell exhaustion including diminished proliferation and impaired cytokine production [22], we hypothesized that PD-1 expression on TRP1-specific T cells may be involved in the maintenance of tolerance. Numerous studies in mice and humans have demonstrated that the PD-1 signaling pathway plays an integral role in the maintenance of exhaustion in both CD4+ and CD8+ T cells in settings of chronic antigen exposure. We found that Ag+GILT-/-Tg mice had a significantly increased percentage of PD-1+ T cells, which were distinct from Treg cells, in comparison to Ag-GILT+/+Tg mice. Blockade of PD-1 signaling partially rescued the ability of CD4+ T cells from Ag+GILT-/-Tg mice to produce IL-2 in response to TRP1 stimulation. These findings demonstrate exposure of melanoma-specific T cells to cognate self antigen in healthy tissue and in the absence of tumor is sufficient to induce an exhaustion-like phenotype characterized by PD-1-mediated immunosuppression.

## Results

### Melanoma-specific T cells that develop in the presence of a self/melanoma antigen lack anti-melanoma and autoimmune activity

**Fig 1:**
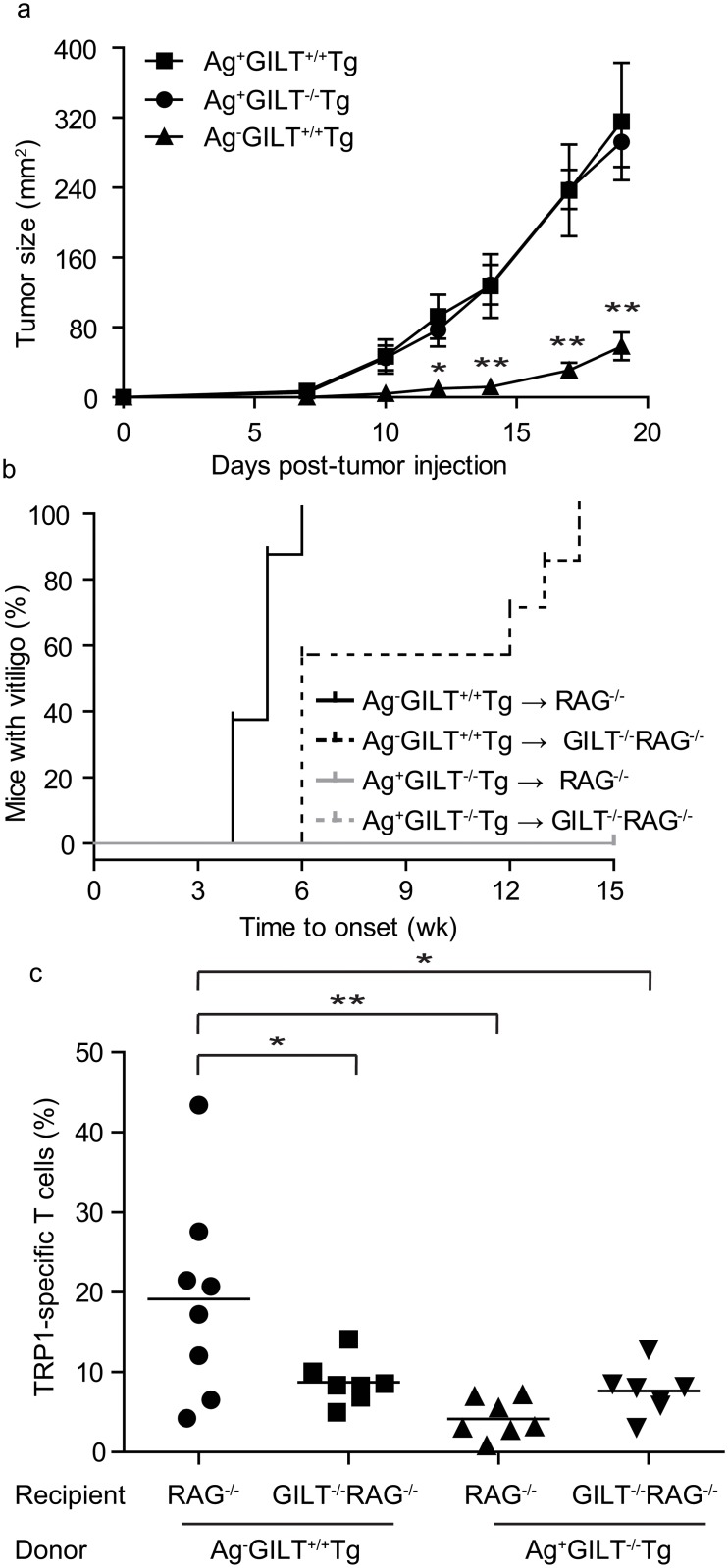
Melanoma-specific T cells that develop in the presence of a self/melanoma antigen lack anti-melanoma and autoimmune activity. **(a)** B16 melanoma cells were subcutaneously injected into Ag+GILT+/+Tg, Ag+GILT-/-Tg and Ag-GILT+/+Tg mice and followed for tumor growth. Data are representative of two independent experiments with five mice per group and compared by ANOVA with the Tukey correction for multiple comparisons (*p<0.05; **p<0.01). **(b)** CD4+ cells from Ag-GILT+/+Tg and Ag+GILT-/-Tg mice were injected into the tail vein of Ag-expressing RAG1-/- or GILT-/-RAG1-/- recipients, and animals were followed for vitiligo onset. Disease onset curves were compared using the log-rank test. The onset of autoimmunity caused by T cells from Ag-GILT+/+Tg mice was delayed in GILT-deficient hosts compared with RAG1-/- hosts (p<0.01). T cells from Ag+GILT-/-Tg mice failed to induce vitiligo in all recipients. **(c)** Fifteen weeks after the adoptive transfer in b, skin-draining lymph node cells from recipient animals were gated on lymphocytes and analyzed for TRP1-specific T cells identified by CD4 and Vβ14 expression. Data are shown from three pooled experiments compared by ANOVA with the Tukey correction for multiple comparisons (*p<0.05; **p<0.01).

To test the anti-melanoma activity of T cells developing in Ag+GILT-/-Tg mice in comparison to Ag-GILT+/+Tg mice and Ag+GILT+/+Tg mice, animals were subcutaneously injected with B16 melanoma cells and followed for tumor growth. The anti-tumor activity of TRP1-specific T cells from Ag-GILT+/+Tg mice has been described previously [19, 21]. Consistent with these studies, melanoma tumor growth was significantly delayed in Ag-GILT+/+Tg mice in comparison to both Ag+GILT-/-Tg and Ag+GILT+/+Tg mice (Fig 1a). Despite the presence of large numbers of peripheral TRP1-specific T cells in Ag+GILT-/-Tg animals [22], there was no impact on melanoma tumor growth in these mice (Fig 1a). Tumor growth in Ag+GILT-/-Tg mice was indiscernible from that seen in Ag+GILT+/+Tg mice, which lack peripheral TRP1-specific T cells [22], indicating that TRP1-specific T cells in Ag+GILT-/-Tg mice lack anti-melanoma activity.

We have previously shown that GILT-deficient antigen presenting cells (APCs) are diminished in their ability to activate TRP1-specific T cells due to reduced presentation of TRP1 [23]. Therefore, it is possible that diminished presentation of TRP1 by APCs in Ag+GILT-/-Tg mice contributes to the lack of anti-tumor T cell activity in these animals. To demonstrate that the lack of anti-melanoma activity in Ag+GILT-/-Tg mice is due to T cell tolerance rather than deficient MHC class II-restricted processing of TRP1 by GILT-deficient APCs, we adoptively transferred CD4+ T cells from Ag-GILT+/+Tg and Ag+GILT-/-Tg mice into Ag-expressing RAG1-/- mice with and without GILT. The recipients were followed for the onset of autoimmune vitiligo for 15 weeks and then skin-draining lymph nodes were analyzed for TRP1-specific T cells. Adoptive transfer of CD4+ T cells from Ag-GILT+/+Tg mice leads to rapid vitiligo in Ag-expressing RAG1-/- recipients with a median onset of five weeks (Fig 1b), consistent with previous findings [22]. In Ag-expressing GILT-/-RAG1-/-recipients, the median onset of vitiligo is significantly delayed by one week (p<0.01; Fig 1b), consistent with a requirement for GILT expression by peripheral APCs for the priming of TRP1-specific T cell responses [23]. In addition, a significantly larger percentage of TRP1-specific T cells were recovered from the skin-draining lymph nodes of GILT-expressing RAG1-/- mice compared to GILT-/-RAG1-/- mice adoptively transferred with CD4+ T cells from Ag-GILT+/+Tg mice (p<0.05; Fig 1c). The delayed onset of autoimmunity following transfer of T cells from Ag-GILT+/+Tg mice into GILT-/-RAG1-/- mice vs. RAG1-/- mice as well as the reduced recovery of TRP1-specific T cells following transfer into GILT-/-RAG1-/- mice highlights the importance of GILT expression in host APC’s for the efficient processing and presentation of TRP1.

Conversely, adoptive transfer of TRP1-specific T cells from Ag+GILT-/-Tg mice failed to induce vitiligo in Ag-expressing RAG1-/- mice with or without GILT (Fig 1b). Furthermore, mice that received T cells from Ag+GILT-/-Tg mice had similar low percentages of TRP1-specific T cells present in the skin-draining lymph nodes regardless of GILT expression in the recipient (Fig 1c). The percentage of TRP1-specific T cells recovered from the skin-draining lymph nodes of RAG1-/- mice adoptively transferred with T cells from Ag+GILT-/-Tg mice was significantly reduced in comparison to RAG1-/- mice adoptively transferred with T cells from Ag-GILT+/+Tg mice (p<0.01; Fig 1c). These data indicate that higher levels of TRP1 presentation in GILT-expressing RAG1-/- mice cannot overcome the dysfunction of T cells from Ag+GILT-/-Tg mice and demonstrate that the unresponsiveness of T cells in Ag+GILT-/-Tg mice is not only due to reduced TRP1 presentation by GILT-deficient APCs. The inability of T cells from Ag+GILT-/-Tg mice to induce vitiligo is not due to reduced T cell numbers as there was no difference between the percentage of TRP1-specific T cells recovered from GILT-/-RAG1-/- mice that received T cells from Ag+GILT-/-Tg mice and GILT-/-RAG1-/- recipients that received T cells from Ag-GILT+/+Tg donors which did develop autoimmunity (Fig 1b and 1c). The failure of T cells from Ag+GILT-/-Tg mice to induce autoimmunity in addition to the similarly reduced recovery of these T cells following adoptive transfer irrespective of GILT expression in the recipient points to an intrinsic defect in T cells from Ag+GILT-/-Tg mice that ultimately impairs their ability to mediate autoimmunity and anti-tumor immunity.

### Treg cell depletion partially rescues the ability of melanoma-specific T cells from TRP1-expressing mice to undergo antigen-specific proliferation

**Fig 2:**
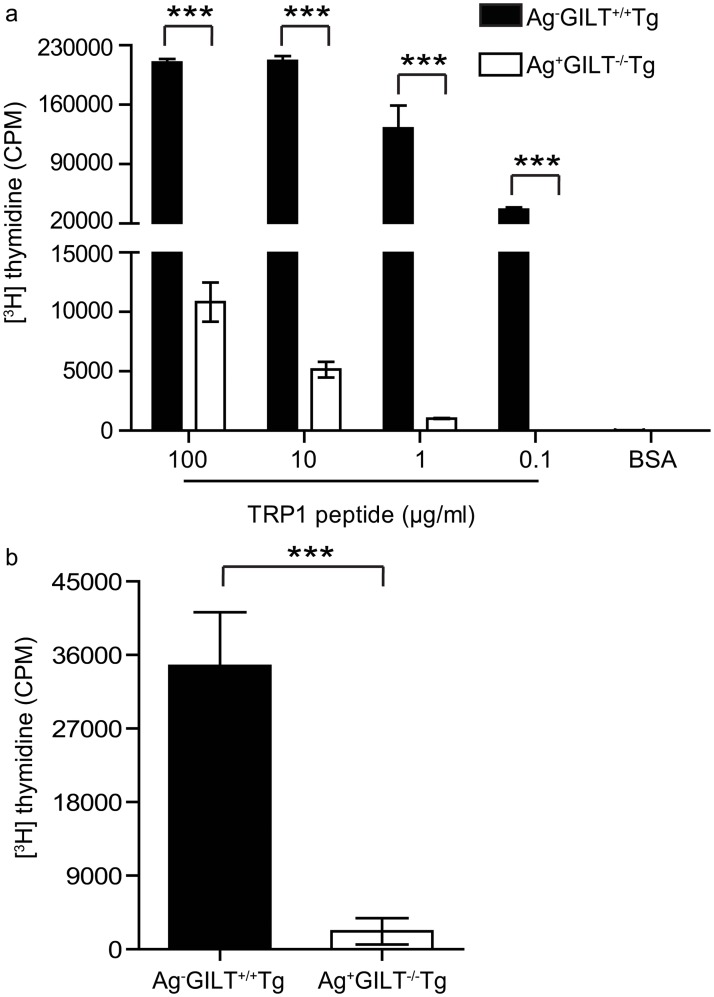
Melanoma-specific T cells from TRP1-expressing mice undergo diminished proliferation in response to TCR stimulation. CD4+ lymph node cells from Ag-GILT+/+Tg and Ag+GILT-/-Tg mice were cocultured with irradiated T cell-depleted splenocytes from wild-type mice and **(a)** TRP1 peptide or **(b)** anti-CD3 and anti-CD28 antibodies for five days. Columns and bars represent means ± standard error of triplicate samples from one experiment. Data are representative of two independent experiments and compared using an unpaired t test (***p<0.001).

TRP1-specific T cells from Ag+GILT-/-Tg mice were recovered in reduced percentages after adoptive transfer in comparison to T cells from Ag-GILT+/+Tg mice, suggesting that T cells from Ag+GILT-/-Tg mice may have impaired antigen-induced proliferation. Therefore, we compared the ability of CD4+ T cells from these two strains to proliferate in response to TRP1. CD4+ T cells from Ag+GILT-/-Tg mice demonstrated markedly diminished TRP1-specific proliferation in comparison to T cells from Ag-GILT+/+Tg animals across a range of peptide concentrations (p<0.001; Fig 2a). In addition, T cells from Ag+GILT-/-Tg mice exhibited diminished proliferation in response to anti-CD3/anti-CD28 stimulation in comparison to T cells from Ag-GILT+/+Tg mice (p<0.001; Fig 2b).

**Fig 3:**
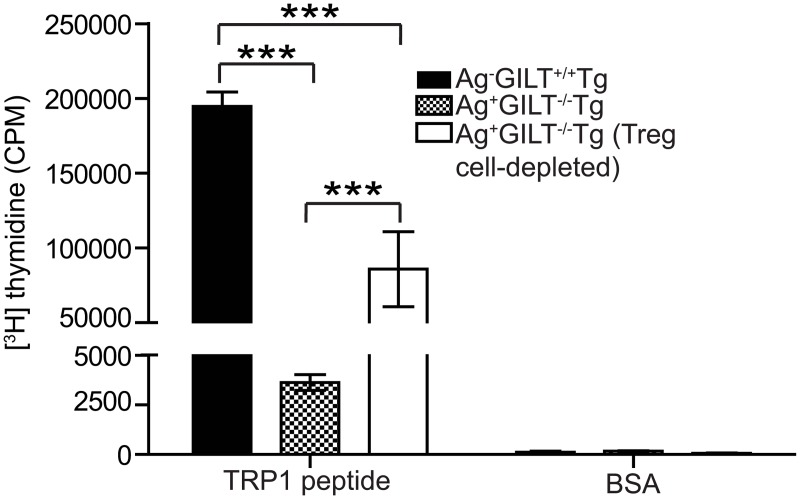
Treg cell depletion partially restores the ability of melanoma-specific T cells from TRP1-expressing mice to proliferate in response to antigen. Total CD4+ and CD4+CD25-T cells were FACS-sorted from Ag+GILT-/-Tg mice and cocultured with irradiated T cell-depleted splenocytes from wild-type mice and TRP1 peptide for five days. Total CD4+ T cells from Ag-GILT+/+Tg mice were included as a positive control. Columns and bars represent means ± standard error of pooled data from two independent experiments compared by ANOVA with the Tukey correction for multiple comparisons (***p<0.001).

We have previously shown that Foxp3+ Treg cells contribute to TRP1-specific T cell tolerance in the Ag+GILT-/-Tg mouse model [22]. To determine if Treg cells in Ag+GILT-/-Tg mice are responsible for the diminished proliferation, we performed TRP1-specific proliferation assays with either Treg cell-depleted or total CD4+ T cells from Ag+GILT-/-Tg animals. The proliferation of total CD4+ T cells from Ag+GILT-/-Tg mice was significantly diminished in comparison to the proliferation of CD4+ T cells from Ag-GILT+/+Tg mice (p<0.001; Fig 3). Treg cell depletion significantly increased the proliferation of T cells from Ag+GILT-/-Tg mice in response to TRP1 peptide stimulation (p<0.001; Fig 3). However, the proliferation of Treg cell-depleted T cells from Ag+GILT-/-Tg mice was still 56% lower than the proliferation of total CD4+ T cells from Ag-GILT+/+Tg mice (p<0.001; Fig 3). These data suggest that the activity of T cells from Ag+GILT-/-Tg mice is constrained by mechanisms in addition to Treg cells.

### An increased percentage of melanoma-specific T cells from TRP1-expressing mice express PD-1

**Fig 4:**
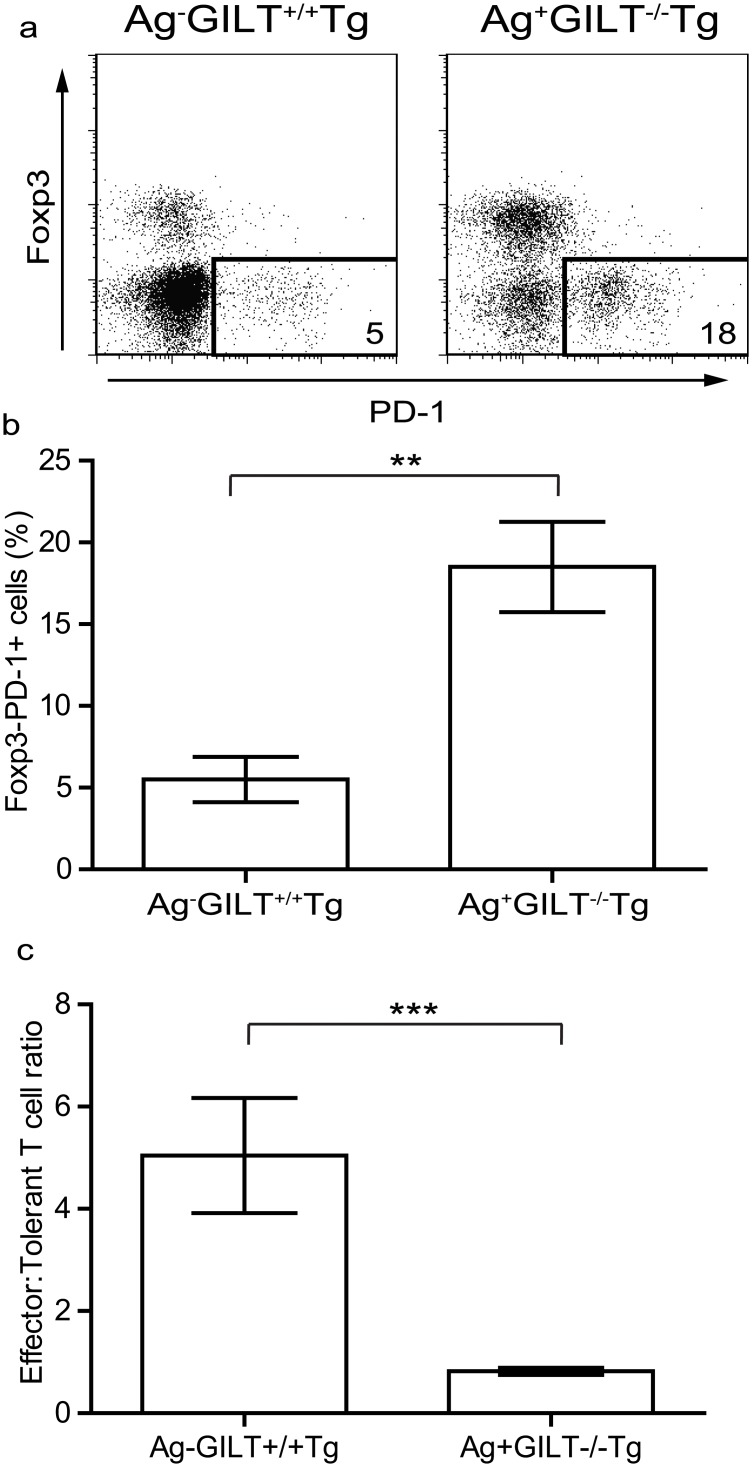
An increased percentage of melanoma-specific T cells from TRP1-expressing mice express PD-1 compared to TRP1-deficient mice. **(a)** Skin-draining lymph node cells from Ag+GILT-/-Tg and Ag-GILT+/+Tg mice were stained and analyzed by flow cytometry. Cells were gated on CD4+Vβ14+ TRP1-specific T cells. **(b)** Percentage of Foxp3-PD-1+ T cells in the CD4+Vβ14+ gate from the skin-draining lymph nodes of Ag-GILT+/+Tg and Ag+GILT-/-Tg mice. **(c)** Ratio of CD4+Vβ14+ TRP1-specific effector (Foxp3-PD-1-) to tolerant (Foxp3+PD-1- and Foxp3-PD-1+) T cells. Data shown in b and c are from two pooled experiments (total n = 6) and are compared using an unpaired t test (**p<0.01; ***p<0.001).

TRP1-specific T cells from Ag+GILT-/-Tg mice display many signs of T cell exhaustion including an antigen-experienced phenotype, impaired cytokine production [22], and diminished proliferation (Figs 2 and 3). Therefore, TRP1-specific T cells from the skin-draining lymph nodes of Ag+GILT-/-Tg and Ag-GILT+/+Tg mice were analyzed for PD-1 expression. PD-1 is an inhibitory member of the CD28 costimulatory molecule superfamily which is involved in peripheral tolerance, T cell exhaustion, and suppression by Treg cells [10, 11]. Engagement of PD-1 with one of its two ligands, programmed death ligand 1 (PD-L1) and PD-L2, leads to the suppression of proliferation and cytokine production [24, 25]. TRP1-specific T cells from Ag+GILT-/-Tg and Ag-GILT+/+Tg mice contained three populations relative to PD-1 and Foxp3 expression, namely Foxp3-PD-1-, Foxp3+PD-1-, and Foxp3-PD-1+ T cells (Fig 4a). Consistent with our earlier report [22], Ag+GILT-/-Tg mice contained a four-fold increase in the percentage of TRP1-specific Foxp3+ Treg cells in comparison to Ag-GILT+/+Tg mice (Fig 4a). Both Ag+GILT-/-Tg and Ag-GILT+/+Tg mice had very few TRP1-specific T cells that express both Foxp3 and PD-1, indicating that Treg cells are not using PD-1 as an inhibitory mechanism in this model (Fig 4a). Ag+GILT-/-Tg mice had a four-fold increase in the percentage of TRP1-specific Foxp3-PD-1+ T cells in their skin-draining lymph nodes in comparison to Ag-GILT+/+Tg mice (p<0.01; Fig 4a and 4b). Furthermore, the ratio of TRP1-specific effector (Foxp3-PD-1-) to tolerant (Foxp3+PD1- and Foxp3-PD-1+) T cells was significantly diminished in Ag+GILT-/-Tg mice to 0.8:1 compared to 5:1 in Ag-GILT+/+Tg mice (p<0.001; Fig 4c). Therefore, increased expression of PD-1 may regulate the activity of TRP1-specific T cells from Ag+GILT-/-Tg animals.

### PD-1 suppresses IL-2 production by melanoma-specific T cells from TRP1-expressing mice

**Fig 5:**
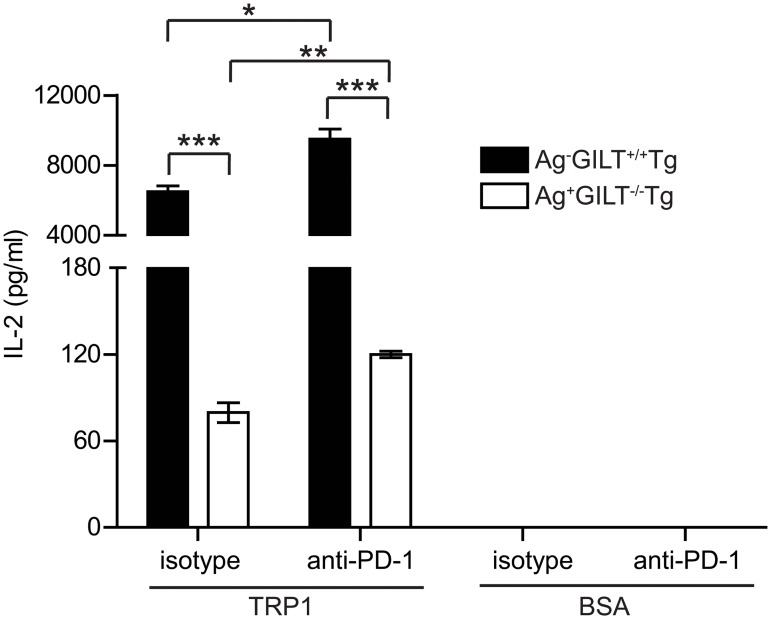
PD-1 suppresses IL-2 production by melanoma-specific T cells from TRP1-expressing mice. CD4+ lymph node cells from Ag-GILT+/+Tg and Ag+GILT-/-Tg mice were cocultured with T cell-depleted splenocytes from wild-type mice and TRP1 peptide or BSA (negative control) with either a PD-1 blocking monoclonal antibody or an isotype control antibody. IL-2 production was measured by ELISA. Columns and bars represent means ± standard error of triplicate samples from one experiment. Data are representative of two independent experiments and are compared by ANOVA with the Tukey correction for multiple comparisons (*p<0.05; **p<0.01; ***p<0.001).

To determine if PD-1 inhibits IL-2 production by TRP1-specific T cells, we performed blocking experiments using a monoclonal antibody to PD-1. IL-2 production by CD4+ T cells from Ag+GILT-/-Tg mice was diminished in comparison to T cells from Ag-GILT+/+Tg animals (p<0.001; Fig 5), consistent with our previous findings [22]. Antibody blockade of PD-1 significantly improved the ability of TRP1-specific T cells from Ag+GILT-/-Tg animals to produce IL-2 in response to antigen by 50% (p<0.01; Fig 5). Similarly, PD-1 blockade resulted in a small increase in IL-2 production by T cells from Ag-GILT+/+Tg mice in response to antigen (p<0.05; Fig 5). The amount of IL-2 produced by T cells from Ag+GILT-/-Tg animals in the presence of PD-1 blockade was still reduced in comparison to T cells from Ag-GILT+/+Tg mice, indicating that PD-1 is partially responsible for the diminished cytokine production (p<0.001; Fig 5). These data demonstrate that PD-1 plays a functional role in modulating T cell cytokine production and contributes to the maintenance of tolerance to TRP1 in our model.

## Discussion

By crossing the TRP1-specific TCR transgenic mouse strain [19] onto the GILT-deficient background, we created Ag+GILT-/-Tg animals in which T cells specific for the melanocyte differentiation antigen TRP1 escape negative selection in the thymus and populate the periphery, but fail to protect from melanoma challenge (Fig 1a) and do not induce autoimmune vitiligo (Fig 1b) [22]. This model has important clinically relevant features in that T cells specific for a self and melanoma antigen develop in the context of endogenous self antigen expression and thus are subject to tolerance mechanisms. Using these animals, we demonstrate that the activity of TRP1-specific T cells is constrained by Treg cells and an exhaustion-like phenotype due to TRP1 expression in healthy tissue and prior to encountering melanoma tumors.

CD4+ T cell dysfunction has been readily described in tumor bearing mouse models and cancer patients and represents a major challenge to the successful application of immunotherapy for cancer. Previous studies in mice have demonstrated that tumor-specific CD4+ T cells can be rendered anergic by exposure to established tumors *in vivo* [8, 9, 26]. The tumor-induced T cell anergy observed in these studies was attributed to suboptimal T cell stimulation by resting APCs which often lack expression of costimulatory molecules. Indeed, activation of APCs in vivo with an antibody to CD40 has been shown to overcome CD4+ T cell anergy in some tumor models [27]. These studies provide important insights into the priming of tumor-specific T cell immunity in a non-inflammatory setting. However, these studies utilize tumor cells engineered to express viral peptides as model tumor antigens which are not subject to self tolerance and may not accurately reflect the immune response to endogenous self/tumor antigens. To address this issue, we developed a mouse model in which T cells specific for an endogenously expressed self/tumor antigen develop.

The decreased percentage of TRP1-specific T cells from Ag+GILT-/-Tg mice after adoptive transfer in RAG1-/- hosts may be due to decreased proliferation, increased apoptosis, or peripheral deletion. Since T cells from Ag+GILT-/-Tg mice adoptively transferred into RAG1-/- recipients with and without GILT were recovered in similar frequencies to T cells from Ag-GILT+/+Tg mice transferred into RAG1-/-GILT-/-recipients (Fig 1c), it is unlikely that T cells from Ag+GILT-/-Tg mice have an increased susceptibility to apoptosis compared to T cells from Ag-GILT+/+Tg mice. Established tumors have been shown to contribute to T cell dysfunction by inducing peripheral deletion of tumor-specific T cells in some models [28]. Peripheral deletion is unlikely to have contributed to the diminished recovery of Ag+GILT-/-Tg T cells seen in our model, because TRP1-specific T cells persisted in all cases. In murine models in which autoreactive T cells are deleted in the periphery by lymph node stromal cells, autoreactive T cells are completely eliminated two weeks after adoptive transfer [29, 30, 31]. In contrast, substantial numbers of CD4+ TRP1-specific T cells from Ag+GILT-/-Tg animals are recovered as late as 15 weeks after transfer (Fig 1c). Therefore, it is most likely that the reduced recovery of Ag+GILT-/-Tg T cells after adoptive transfer is due to impaired T cell proliferation. Indeed, we show that TRP1-specific T cells from Ag+GILT-/-Tg mice undergo diminished proliferation *in vitro* in comparison to T cells from Ag-GILT+/+Tg mice (Fig 2).

T cell exhaustion has also been shown to induce T cell dysfunction in tumor-bearing hosts [32]. Under conditions of chronic antigen exposure, persistent T cell stimulation has been shown to induce a state of exhaustion where T cells gradually lose vital effector functions including proliferation and cytokine production [32]. Ag+GILT-/-Tg mice express TRP1, and we have previously shown that GILT-deficient APCs are capable of presenting low levels of TRP1 [23]. Additionally, a portion of TRP1-specific T cells from Ag+GILT-/-Tg mice express low levels of CD62L and high levels of CD44 [22], indicating prior exposure to their cognate antigen. The exhausted phenotype of TRP1-specific T cells that develop in Ag+GILT-/-Tg mice is further supported by diminished proliferation (Fig 2), diminished cytokine production (Fig 5) [22], high levels of PD-1 expression, which contributes to diminished cytokine production (Figs 4 and 5), and the failure to protect from melanoma challenge and induce vitiligo (Fig 1) [22].

The potent ability of inhibitory immune checkpoint receptors like PD-1 to tolerize tumor-specific T cells has recently been demonstrated in several tumor models and blockade of the PD-1 signaling pathway has shown promise in rescuing the effector functions of exhausted T cells in the context of cancer [10, 11]. We found that blockade of PD-1 signaling using a PD-1 blocking antibody partially restored the ability of T cells from Ag+GILT-/-Tg mice to produce IL-2 in response to TRP1 stimulation *in vitro* (Fig 5). However, the amount of IL-2 produced by TRP1-specific T cells from Ag+GILT-/-Tg mice in the presence of a PD-1 blocking antibody was still significantly reduced in comparison to T cells from Ag-GILT+/+Tg mice (Fig 5), suggesting that PD-1 blockade alone may not be sufficient to completely restore the effector function of melanoma-specific T cells. These findings are consistent with several recent studies demonstrating that PD-1 blockade alone is unable to fully rescue anti-tumor immune function in murine models. For example, in mice bearing recurrent B16 melanoma tumors, full tumor regression could only be achieved when anti-PD-L1 treatment was combined with Treg cell depletion or blockade of the inhibitory receptor LAG-3 [33]. Similarly, combined blockade of PD-1 and LAG-3 was required to achieve full tumor regression in murine models of fibrosarcoma and colon cancer [34]. In a murine model of colon cancer, blockade of both PD-1 and TIM-3 resulted in more effective anti-tumor immunity than blockade of either pathway alone [35]. Furthermore, anti-TIM-3 treatment synergized with inhibition of PD-1 signaling to restore the effector functions of tumor-specific T cells from melanoma patients in *ex vivo* stimulation studies [16]. A recent phase 1 study in melanoma patients, suggesting that combination therapy with ipilimumab and nivolumab may result in superior clinical activity compared to monotherapy, further supports blockade of multiple inhibitory immune checkpoint pathways to achieve optimal anti-tumor efficacy [36].

Using the TRP1-specific TCR transgenic mouse model, we have identified critical determinants involved in peripheral tolerance to a self and melanoma antigen. Exposure of melanoma-specific T cells to cognate self antigen in healthy tissue and in the absence of tumor is sufficient to induce Treg cells and an exhaustion-like phenotype characterized by PD-1-mediated immunosuppression. These findings have important implications for immunotherapy of melanoma and cutaneous autoimmunity as targeting multiple tolerance pathways will likely enhance therapeutic efficacy.

## Methods

### Cell lines and mice

The B16.F10 murine melanoma cell line was purchased from the American Type Culture Collection (ATCC, Manassas, VA). C57BL/6J (wild-type) and RAG1-/- mice were obtained from Jackson Laboratory (Bar Harbor, ME). GILT-/- mice were provided by Dr. Peter Cresswell [37]. TCR transgenic (GILT+/+ RAG1-/- TRP1-specific TCR transgenic) mice with and without TRP1 were provided by Dr. Nicholas Restifo [19]. Ag+GILT-/-Tg (TRP1-expressing GILT-/- RAG1-/- TRP1-specific TCR transgenic) mice have been described previously [22]. Thymuses, spleens, and skin-draining lymph nodes were isolated as described [23]. All animals were housed in microisolator cages. This study was carried out in strict accordance with the recommendations in the Guide for the Care and Use of Laboratory Animals of the National Institutes of Health. The protocols were approved by the University of Arizona’s Institutional Animal Care and Use Committee (Protocol Number: 13–469) and all efforts were made to minimize suffering.

### Flow cytometry

Cells were stained with FITC, phycoerythrin, phycoerythrin-Cy7, PerCP, or APC-conjugated mAbs against murine Vβ14 (clone 14–2), CD4 (RM4-5), CD25 (PC61.5), Foxp3 (FJK-16s), PD-1 (J43) and corresponding isotype controls (BD Biosciences, San Jose, CA; eBioscience, San Diego, CA), as described [23]. For intracellular staining of Foxp3, cells were fixed and permeabilized using the Foxp3 staining buffer set (eBioscience). Cell analysis was performed using an LSRII, and cell sorting was performed using a FACS Aria-II (BDBiosciences).

### Tumor challenge

Six-week-old male Ag-GILT+/+Tg, Ag+GILT-/-Tg, and Ag+GILT+/+Tg mice (n = 5 per group) were injected subcutaneously with B16.F10 tumor cells (2.5x10^5^) in 100 μl PBS into the right flank on day 0. Tumor growth was monitored three times weekly by measuring tumor length (L) and width (W), and tumor size (mm^2^) was calculated using the formula L × W.

### Adoptive transfer

CD4+ TRP1-specific T cells were isolated from pooled lymph node and spleen cells from Ag-GILT+/+Tg and Ag+GILT-/-Tg mice using the EasySep mouse CD4 positive selection kit (Stemcell Technologies, Vancouver, Canada). CD4+ T-cell purity was 90–95%.CD4+ T cells (2.5x10^5^) were injected intravenously into each RAG1-/- mouse. Fifteen weeks after transfer, skin-draining lymph node cells were isolated and analyzed for CD4 and Vβ14 expression.

### *In vitro* proliferation assay

CD4+ TRP1-specific T cells (1x10^5^) were positively selected as described above or, for Treg cell depletion, total CD4+ and CD4+CD25- T cells were FACS-sorted from Ag+GILT-/-Tg pooled lymph node and spleen cells. The purity of sorted CD4+CD25-Tcells was >95%. TRP1-specific T cells were cocultured for 5 days with irradiated (3000 rad) T cell-depleted wild type spleen cells and murine TRP1_109–130_ peptide NCGTCRPGWRGAACNQKILTVR or BSA. T cells were depleted using the EasySep mouse CD90 positive selection kit (Stemcell Technologies). Some T cells were stimulated with plate-bound anti-CD3ε (145-2C11;10 μg/ml) and soluble anti-CD28 (37.51; 2 μg/ml). [^3^H]thymidine was added for the final 18 hours of culture.

### *In vitro* PD-1 blockade

Positively selected CD4+ TRP1-specific T cells (1x10^5^) were cocultured for 48 hours with 5x10^5^ T cell-depleted wild-type spleen cells and murine TRP1_109-130_ peptide (10 μg/mL) or BSA (1mg/ml) with either PD-1 blocking antibody (5μg/ml) or isotype control antibody (5μg/ml). The IL-2 concentration in culture supernatants was determined by ELISA (BD Biosciences).
